# Early heme oxygenase 1 induction delays tumour initiation and enhances DNA damage repair in liver macrophages of *Mdr2*^−/−^ mice

**DOI:** 10.1038/s41598-018-33233-0

**Published:** 2018-11-02

**Authors:** Roja Barikbin, Laura Berkhout, Julia Bolik, Dirk Schmidt-Arras, Thomas Ernst, Harald Ittrich, Gerhard Adam, Ann Parplys, Christian Casar, Till Krech, Khalil Karimi, Gabriele Sass, Gisa Tiegs

**Affiliations:** 1 Institute of Experimental Immunology and Hepatology, University Medical Center Hamburg-Eppendorf, Hamburg, Germany; 2 Institute of Biochemistry, Christian-Albrechts-University Kiel, Kiel, Germany; 3 Department of Diagnostic and Interventional Radiology, University Medical Center Hamburg-Eppendorf, Hamburg, Germany; 4 Erwin L. Hahn Institute for Magnetic Resonance Imaging, University Duisburg-Essen, Duisburg, Germany; 5 Department of Radiotherapy and Radio-Oncology, University Medical Center Hamburg-Eppendorf, Hamburg, Germany; 6 Medical Clinics I, University Medical Center Hamburg-Eppendorf, Hamburg, Germany; 7 Institute of Pathology, University Medical Center Hamburg-Eppendorf, Hamburg, Germany; 8 Department of Pathobiology, University of Guelph, Guelph, ON Canada; 9 Department of Infectious Diseases, California Institute for Medical Research, San Jose, CA USA

## Abstract

Multi drug resistance protein 2 knockout mice (*Mdr2*^−/−^) are a mouse model of chronic liver inflammation and inflammation-induced tumour development. Here we investigated the kinetics of early heme oxygenase 1 (HO-1) induction on inflammation, tumour development, and DNA damage in *Mdr2*^−/−^ mice. HO-1 was induced by intraperitoneal injection of cobalt protoporphyrin IX (CoPP) twice weekly for 9 consecutive weeks. Immediately after HO-1 induction, liver function improved and infiltration of CD4^+^ and CD8^+^ T cells was reduced. Furthermore, we observed increased p38 activation with concomitant reduction of *Cyclin D1* expression in aged *Mdr2*^−/−^ mice. Long-term effects of HO-1 induction included increased CD8^+^ T cell infiltration as well as delayed and reduced tumour growth in one-year-old animals. Unexpectedly, DNA double-strand breaks were detected predominantly in macrophages of 65-week-old *Mdr2*^−/−^ mice, while DNA damage was reduced in response to early HO-1 induction *in vivo* and *in vitro*. Overall, early induction of HO-1 in *Mdr2*^−/−^ mice had a beneficial short-term effect on liver function and reduced hepatic T cell accumulation. Long-term effects of early HO-1 induction were increased CD8^+^ T cell numbers, decreased proliferation as wells as reduced DNA damage in liver macrophages of aged animals, accompanied by delayed and reduced tumour growth.

## Introduction

Chronic inflammation of the liver, e.g. by hepatitis B or C virus infections, initiates wound healing processes and immune responses which promote development of hepatocellular carcinoma (HCC)^1,2^. During the course of chronic inflammation, permanently activated regenerative processes increase the risk of mutations, as well as chromosomal aberrations and DNA damage^3^. *Mdr2*^−/−^ mice are a widely used model of inflammation-induced HCC. These animals lack the multi drug resistance protein 2 (MDR2), a phosphatidylcholine transporter, resulting in dysfunctional phospholipid secretion and increased oxidative stress, which in turn leads to chronic liver damage, leukocyte infiltration, fibrosis, and over time to HCC development^4,5^.

Immune cells mediate pro- or anti-carcinogenic effects depending on cell type and environment. Myeloid cells for example, can promote carcinogenesis through regulation of senescence, angiogenesis, extracellular matrix remodelling, or metastasis formation^6^. Furthermore, a majority of HCC patients display defects in dendritic cell (DC) maturation, which eventually impairs T cell responses^7^. Alterations of the adaptive immune response in HCC patients include a reduced CD4^+^ T cell response and impaired CD8^+^ T cell function^8,9^.

The heme oxygenase 1 (HO-1) enzyme catabolizes the oxidative degradation of heme to carbon monoxide (CO), free iron, and biliverdin^10^. HO-1 and its products exert anti-inflammatory, anti-viral, anti-apoptotic, and anti-proliferative functions^11^. The impact of HO-1 function on tumour development is controversially discussed. While overexpression of HO-1 decreased cell proliferation and invasion in a prostate cancer mouse model^12^, silencing of HO-1 reduced tumour growth in an orthotopic liver tumour mouse model^13^. In contrast, the anti-inflammatory effect of HO-1 function is commonly accepted. We previously showed that induction or overexpression of HO-1 attenuates acute liver inflammation^14,15^. We also demonstrated that early induction of HO-1 in *Mdr2*^−/−^ mice interferes with inflammation, fibrosis, and dysplastic nodule formation^16^. Furthermore, HO-1 reduces tissue damage by suppressing the activation and cytokine production of heme sensing macrophages^17^, polymorphonuclear cells^18^, and DCs^18–20^. HO-1 activity inhibits T cell activation in various mouse models, including promotion of T cell tolerance against transplanted organs^19^. Whether HO-1 attenuates inflammation and subsequent tumour development, or promotes adverse effects such as suppression of tumour-specific immune responses appears to highly depend on the time point of induction.

The present study investigated short- and long-term effects of early HO-1 induction on liver function, proliferation, DNA damage, DNA damage repair (DDR) and tumour growth in *Mdr2*^−/−^ mice. Next to short-term effects of early HO-1 induction on liver damage and T cell infiltration, we observed a lasting downregulation of *Cyclin D1* expression most likely via p38 activation. We also found severe DNA damage especially in hepatic macrophages of aged *Mdr2*^−/−^ mice, which was significantly reduced in CoPP treated animals. In addition, bone marrow-derived macrophages (BMDMs) from Mdr2^−/−^ mice, in contrast to WT and CoPP treated Mdr2^−/−^ mice, failed to induce HO-1 as a stress response upon DNA damage. CoPP treatment attenuated DNA damage *in vitro* and *in vivo* as well as it reduced phagocytic activity of macrophages, which suggests reduced oxidative stress in hepatic and BM derived macrophages as a consequence of HO-1 induction. Taken together, early HO-1 induction interfered with several inflammatory and tumour promoting processes.

## Results

### Long-term effects of early HO-1 induction in *Mdr2*^−/−^ mice

**Figure 1:**
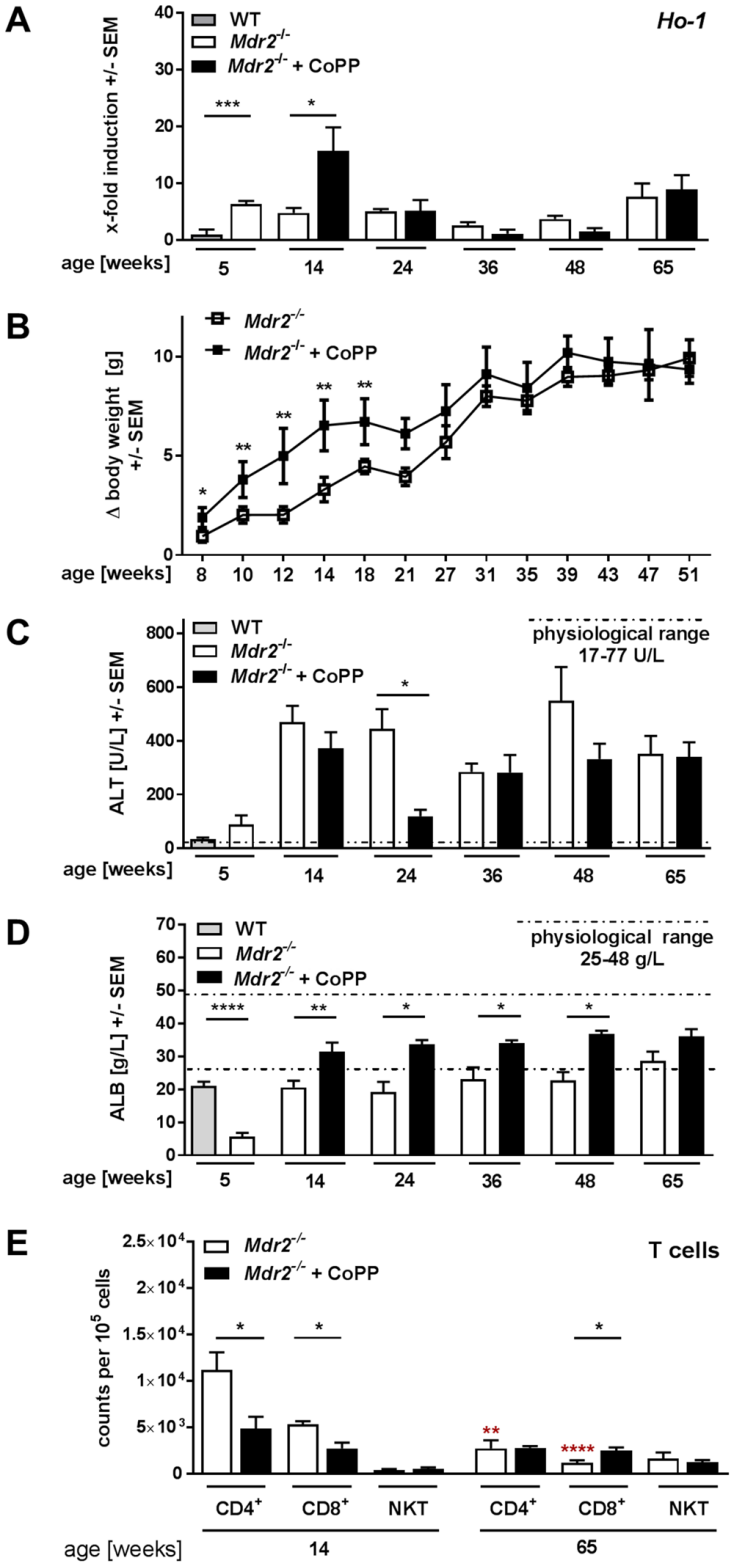
Long-term effects of early HO-1 induction in *Mdr2*^−/−^ mice. (**A**) Hepatic *Ho-1* expression over time of *Mdr2*^−/−^ treated with either HO-1 inducer CoPP [5 mg/kg] or PBS twice a week for nine consecutive weeks (week 5–14), compared to 5-week-old WT mice (n ≥ 4). (**B**) Body weight gain in relation to initial weight over time of two representative experiments with mice described in (**A**) (n ≥ 7). (**C**) Activity of plasma alanine aminotransferase (ALT) over time of mice described in (**A**) (n ≥ 6). (**D**) Plasma level of albumin of mice described in (**A**) (n ≥ 6). The dotted lines indicate physiological range of plasma ALT (17–77 U/L) and ALB (24–48 g/L) of WT animals. (**E)** Hepatic CD4^+^ T cells, CD8^+^ T cells, and NKT cells of 14- and 65-week-old animals. Data are expressed as means ± SEM. *P ≤ 0.05, **P ≤ 0.01, ***P ≤ 0.001, ****P ≤ 0.0001. Asterisks in red depict significance of difference between 14- and 65-week old PBS treated *Mdr2*^−/−^ mice.

To investigate long-term effects of early HO-1 induction, *Mdr2*^−/−^ mice were treated with either PBS or CoPP for 9 weeks (week 5–14) and sacrificed for analysis at different time points throughout a period of 65 weeks (Supplementary Fig. 1A). Analysis of *Ho-1* mRNA expression confirmed successful induction of HO-1 in 14-week-old *Mdr2*^−/−^ mice treated with CoPP (Fig. 1A). Subsequently, the *Ho-1* mRNA expression did not differ between control and CoPP treated *Mdr2*^−/−^ mice. However, CoPP treated mice displayed a significantly higher body weight gain than control treated animals up to one month after termination of treatment, indicating an improved overall health (Fig. 1B). PBS treated *Mdr2*^−/−^ mice presented with increasing liver damage over time, demonstrated by rising plasma levels of ALT, which was only marginally reduced in CoPP treated animals (Fig. 1C). The strongest effect of CoPP treatment was seen in 24-week-old animals. However, plasma levels of albumin were significantly elevated in CoPP treated *Mdr2*^−/−^ mice until week 48, indicating improved liver function in response to HO-1 induction (Fig. 1D). Flow cytometric analysis (Fig. 1E; gating strategy in Supplementary Fig. 1B) revealed significantly decreased CD4^+^ and CD8^+^ T cell infiltration into the injured liver of 14-week-old CoPP treated mice compared to PBS treated mice. Aged PBS treated *Mdr2*^−/−^ mice displayed strongly decreased numbers of CD4^+^ T cells compared to the 14 week-old mice. The CoPP treated mice on the other hand showed only a slight reduction of CD4^+^ T cells over time and the same number of cytotoxic CD8^+^ T cells at 65 weeks of age. Since CD8^+^ T cells are known to play an integral part of the anti-tumour response^9^ and were decreased in 65-week-old PBS treated *Mdr2*^−/−^ mice (Fig. 1E), the significantly increased number of CD8^+^ T cells in aged *Mdr2*^−/−^ mice treated with CoPP may indicate a less tumour susceptible microenvironment.

### Early HO-1 induction delays tumour development in *Mdr2*^−/−^ mice

**Figure 2:**
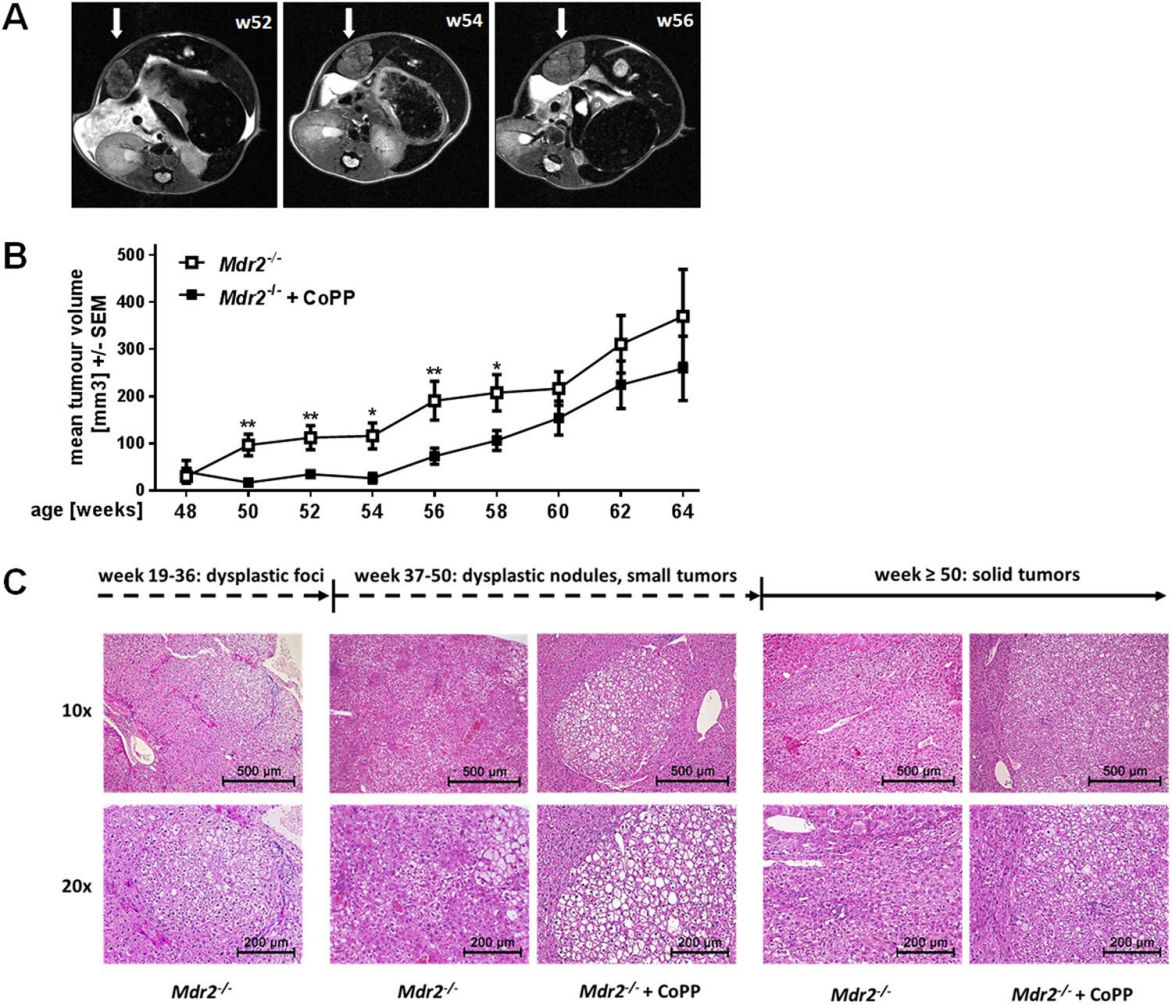
Early induction of HO-1 in *Mdr2*^−/−^ mice delays tumour development. (**A**) Representative images of MRI scans of an *Mdr2*^−/−^ mouse at the age of 52, 54, and 56 weeks. Arrows indicate tumorous tissue. (**B**) Mean tumour volume of *Mdr2*^−/−^ mice treated with either PBS (n = 39) or CoPP (n = 48) as described in Suppl. Fig. 1A, analysed with MR imaging from week 48 to week 64. (**C**) H&E stained tissue sections of dysplastic foci, dysplastic nodules and tumour tissue of *Mdr2*^−/−^ mice treated with either PBS or CoPP as described in Suppl. Fig. 1A. Scale and magnification are indicated. Data are expressed as means ± SEM. *P ≤ 0.05, **P ≤ 0.01.

Chronic hepatic inflammation and fibrosis in *Mdr2*^−/−^ mice results in tumour development within approximately one year of age^5^. Long-term effects of early HO-1 induction on tumour development were monitored by magnetic resonance imaging (MRI). The cumulative tumour volume was measured for each mouse at different time points, starting from the first appearance of dysplastic nodules (48 weeks) until the mice were sacrificed at 65 weeks; an exemplary image sequence of MRI scans over time is presented in Fig. 2A. CoPP treated animals showed significantly decreased mean tumour volumes from week 50 to 58 compared to control treated mice (Fig. 2B). Histological analysis of dysplastic nodules in younger mice (up to 50 weeks) as well as of tumour tissue from older mice (50+ weeks) (Fig. 2C) revealed no phenotypical differences of malignancies derived from PBS and CoPP treated animals. This is probably due to the formation of numerous tumours per animal along with a great variance in histomorphology. PBS as well as CoPP treated animals displayed tumours with varying morphology, vascularization, fat content and differentiation state. Nevertheless, early HO-1 induction successfully delayed malignant growth for over 2 months.

### Early induction of HO-1 in *Mdr2*^−/−^ mice may interfere with hepatocellular proliferation

**Figure 3:**
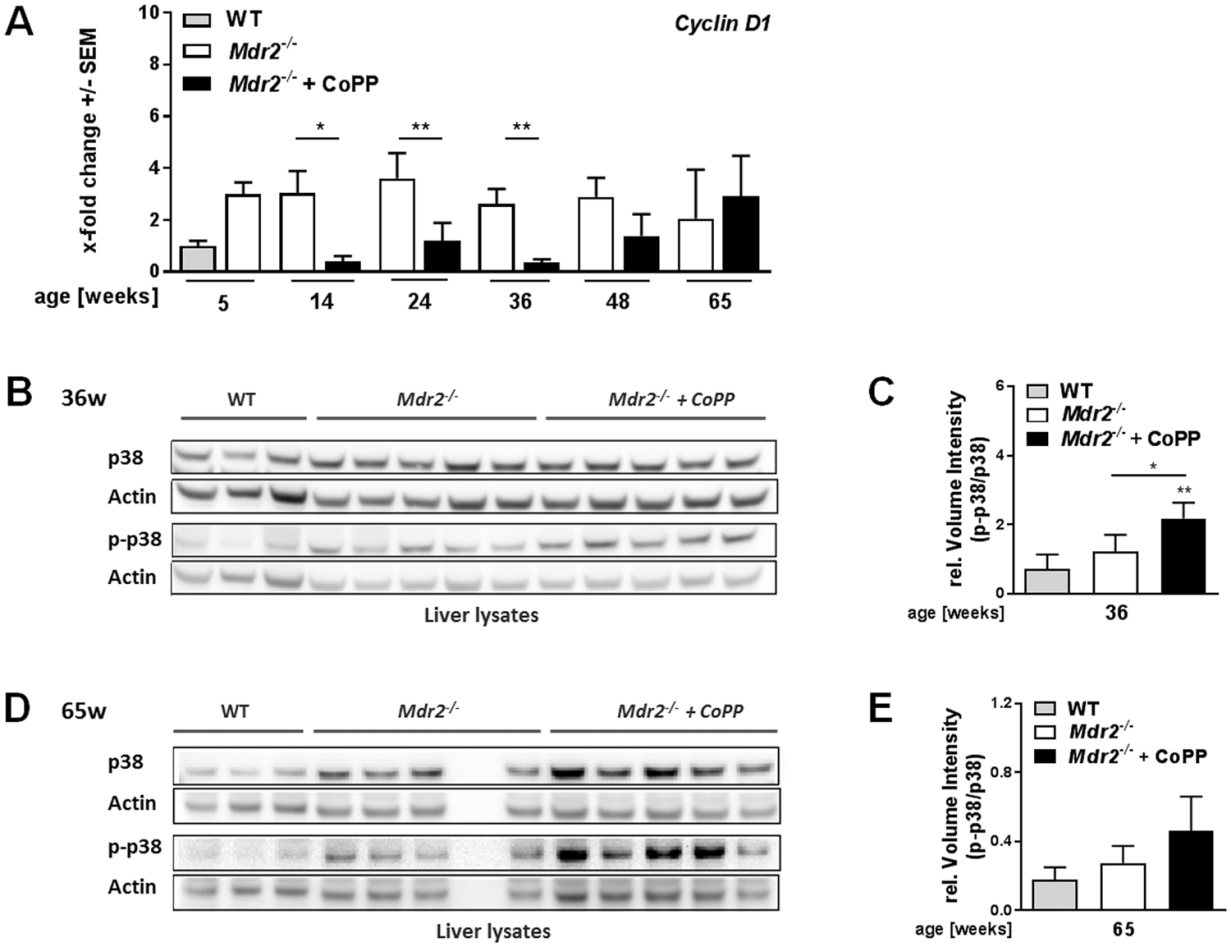
Early induction of HO-1 in *Mdr2*^−/−^ mice may interfere with hepatocellular proliferation. (**A**) Hepatic mRNA expression levels of *Cyclin D1* determined by quantitative real time RT-PCR in the livers of *Mdr2*^−/−^ mice treated with either PBS or CoPP as described in Suppl. Fig. 1A, normalized to 5-week-old WT  mice (n ≥ 5). (**B**) Hepatic protein expression level and phosphorylation state of p38 determined by western blot (WB) analysis of 36-week-old *Mdr2*^−/−^ mice treated as described in Suppl. Fig. 1A compared to WT mice of the same age (n ≥ 3). (**C**) Quantification of the WB described in (**B**). (**D**) Hepatic protein expression level and phosphorylation state of p38 determined by western blot (WB) analysis of 65-week-old *Mdr2*^−/−^ mice treated as described in Suppl. Fig. 1A compared to WT mice of the same age (n ≥ 3). (**E**) Quantification of the WBs described in (**D**). The samples for the p38 and p-p38 WBs were derived from the same experiment and gels/blots were processed in parallel. Images of the full length blots are presented in Supplementary Fig. 4. Data are expressed as means ± SEM. *P ≤ 0.05, **P ≤ 0.01, ***P ≤ 0.001, ****P ≤ 0.0001.

Tumour development is driven by cell cycle activation and tumour cell proliferation which is regulated by cyclins such as Cyclin D1^21,22^. We found previously that HO-1induction reduces *Cyclin D1* mRNA expression in young *Mdr2*^−/−^ mice (12 weeks) treated with CoPP^16^. Here we show that early HO-1 induction resulted in sustained suppression of *Cyclin D1* expression over months after termination of CoPP treatment (Fig. 3A). Furthermore, we found increased protein expression and phosphorylation of p38 (p-p38; Fig. 3B,C) a known negative regulator of *Cyclin D1* expression^23^ in older mice (36 weeks). Interestingly, in 65-week-old animals the p-p38/p38 ratio is still elevated (Fig. 3D,E). These findings suggest that early HO-1 induction has a long-term effect on MAP-Kinase signalling, which may regulate *Cyclin D1* mediated proliferation.

### HO-1 induction reduces DNA damage in macrophages *in vitro* and *in vivo*

**Figure 4:**
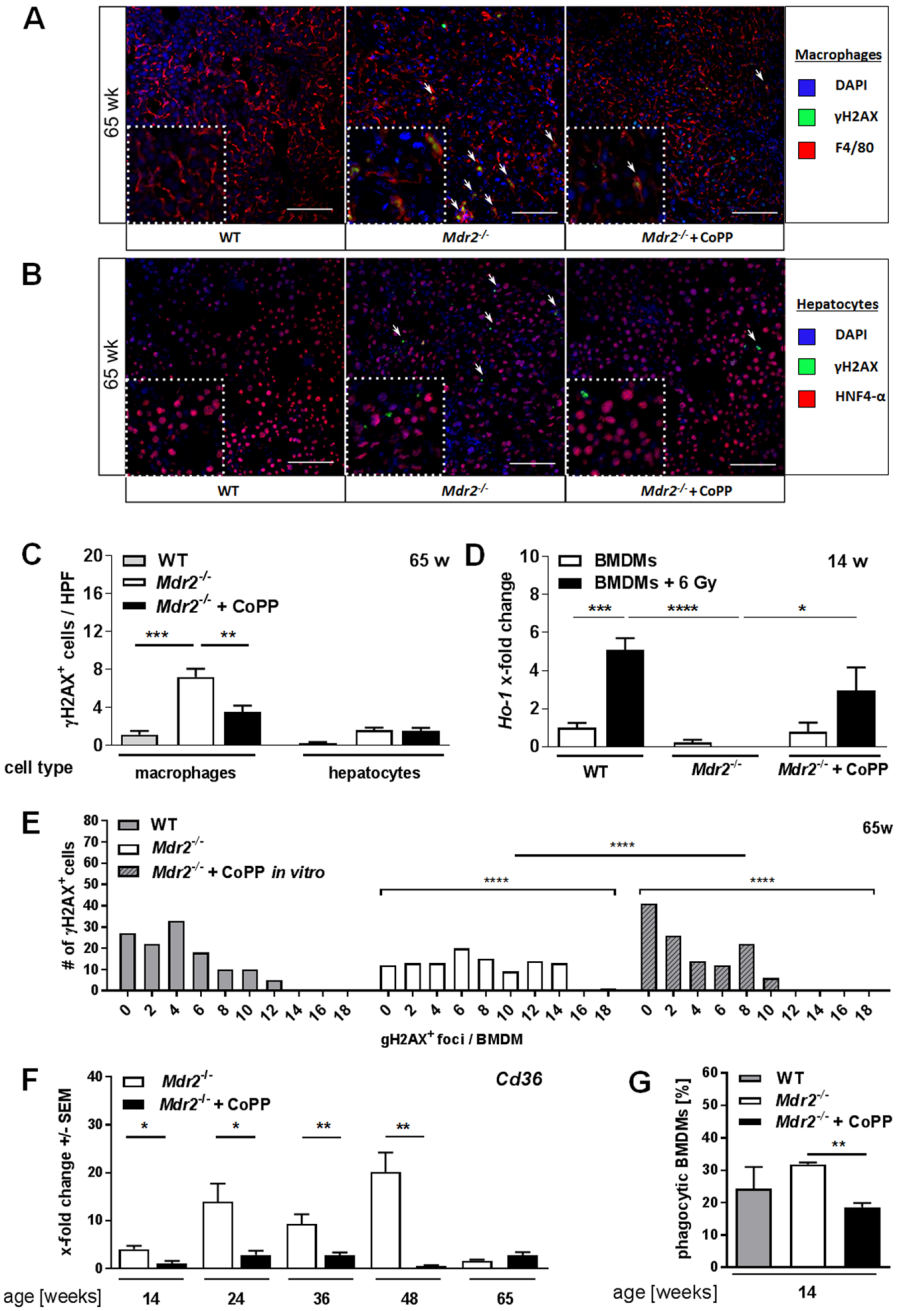
HO-1 induction reduces DNA damage in macrophages *in vitro* and *in vivo*. Representative images (20x) of tissue sections of 65-week-old *Mdr2*^−/−^ mice, treated as described in Suppl. Fig. 1A and WT mice of the same age stained for (**A**) DAPI, γH2AX, and the macrophage marker F4/80 as well as (**B**) DAPI, γH2AX, and the hepatocyte marker HNF4-alpha. (**C**) Quantification of γH2AX^+^ F4/80^+^ macrophages and γH2AX^+^ HNF4-alpha^+^ hepatocytes in tissue sections described in A (n ≥ 4 HPF/slide). (**D**) *Ho-1* mRNA expression levels of BMDMs derived from 14-week-old WT and *Mdr2*^−/−^ mice, with or without irradiation, with or without CoPP treatment [10 µg/ml; 24 h prior to irradiation] of one representative experiment (n = 3). (**E**) Frequency distribution of γH2AX^+^ foci in BMDMs (65w; WT, *Mdr2*^−/−^) with or without CoPP treatment *in vitro* [10 µg/ml; 24 h]. (**F**) Hepatic mRNA expression levels of *Cd36* determined by quantitative real time RT-PCR in livers of mice described in Suppl. Fig. 1A. (**G**) Quantification of phagocytic activity of BMDMs derived from 14-week-old animals described in Suppl. Fig. 1A determined by flow cytometry (n ≥ 3; one representative experiment). Data expressed as means ± SEM. *P ≤ 0.05, **P ≤ 0.01, ***P ≤ 0.001, ****P ≤ 0.0001.

Malignant transformation is associated with accumulation of mutations, DNA damage and altered capacity of DDR over time^3^. We therefore investigated DNA damage in *Mdr2*^−/−^ mice by the assessment of γH2AX^+^ cells in liver tissue sections of aged animals. γH2AX^+^ nuclei were present in F4/80^+^ macrophages or HNF4-α^+^ hepatocytes (Fig. 4A,B) in tissue sections of 65-week-old WT and *Mdr2*^−/−^ mice. While we observed DNA damage in few hepatocytes, γH2AX^+^ nuclei were predominantly found in F4/80^+^ macrophages in *Mdr2*^−/−^ mice. Interestingly, CoPP-treated *Mdr2*^−/−^ mice displayed significantly reduced numbers of γH2AX^+^ macrophages compared to control treated animals (Fig. 4C). By contrast, HNF4α^+^ hepatocytes did not show a significant change in DNA damage occurrence upon CoPP treatment.

To verify that DNA damage accumulated in macrophages rather than hepatocytes, we stained tissue sections of 14- as well as 36-week-old *Mdr2*^−/−^ mice (Supplementary Fig. 2A,B) and additionally performed western blot analysis of isolated hepatic macrophages and hepatocytes from these mice (Supplementary Fig. 2C). In both cases we observed little overall DNA damage in 14-week-old Mdr2^−/−^ mice, which increased with age (36-week-old animals) in macrophages and to a much smaller extent also in the hepatocytes.

In order to investigate the functional consequences of DNA damage in macrophages, we induced DNA double-strand breaks (DBS) in BMDMs of WT and *Mdr2*^−/−^ mice (PBS vs CoPP treated [10 µg/ml; 24 h]) by gamma irradiation [6 Gy]. WT macrophages were able to significantly induce *Ho-1* expression upon irradiation (Fig. 4D), whereas *Mdr2*^−/−^ mice derived BMDMs displayed a reduced basal expression of *Ho-1* and failed to induce it as a stress response to irradiation. *Mdr2*^−/−^ mice derived BMDMs pre-treated with CoPP *in vitro* showed increased *Ho-1* expression compared to untreated *Mdr2*^−/−^ BMDMs and further increased its expression upon irradiation. We also investigated the basal DNA damage in BMDMs from 14-week-old (Supplementary Fig. 2D) and 65-week-old (Fig. 4E) WT or *Mdr2*^−/−^, mice with or without CoPP treatment *in vitro* and *in vivo* (14-week-old). A frequency distribution of γH2AX^+^ foci per cell illustrates significant differences between WT and *Mdr2*^−/−^, as well as CoPP treated vs. control treated *Mdr2*^−/−^ -derived BMDMs. BMDMs derived from 65-week-old *Mdr2*^−/−^ mice showed a significantly higher number of γH2AX^+^ foci per cell compared to WT derived BMDMs. HO-1 induction for 24 h *in vitro* significantly decreased the number of γH2AX^+^ foci per cell in *Mdr2*^−/−^ mice derived BMDMs (Fig. 4E). BMDMs isolated from 14-week-old *Mdr2*^−/−^ mice confirmed a similar reduction of γH2AX^+^ foci per cell when mice were treated with CoPP *in vivo* (week 5–14) to those treated *in vitro* (CoPP treatment for 24 h) (Supplementary Fig. 2D).

A further indicator of altered macrophage function in response to early HO-1 induction was seen in the significantly decreased hepatic expression of *Cd36* in CoPP treated animals throughout the first 48 weeks of life (Fig. 4F). As CD36 can mediate phagocytosis of macrophages^24^, we further investigated the phagocytic capacities of BMDMs from WT and *Mdr2*^−/−^ mice. BMDMs derived from *Mdr2*^−/−^ mice, showed a significantly reduced phagocytic activity after HO-1 induction (CoPP [10 µg/ml] for 24 h) (Fig. 4G, gating strategy in Supplementary Fig. 1C, representative dot plots are presented in Supplementary Fig. 3). These findings imply that early HO-1 induction has a persistent effect on DNA integrity and phagocytic activity of macrophages in *Mdr2*^−/−^ mice.

## Discussion

Chronic liver inflammation is a major health issue and often associated with HCC development, which is the second most frequent cause of cancer related death worldwide^25^. Apart from surgical procedures and irradiation, only the multikinase inhibitor sorafenib is known to prolong survival of advanced HCC patients for about 3 months^26^. A better understanding of immune mediated processes and molecular changes during tumour development is therefore essential to improve treatment options for HCC patients.

We showed previously, that HO-1 induction in *Mdr2*^−/−^ mice not only reduced inflammation and subsequent fibrosis, but also induced fibrolysis and attenuated the amount of dysplastic nodules in female *Mdr2*^−/−^ mice up to 19 weeks of age^16^. Here, we monitored short- and long-term effects of HO-1 induction (week 5–14) until one year after termination of treatment. Early induction of HO-1 had broad beneficial effects on the overall health. The improved body weight gain and albumin release are strong indicators of an improved health status and liver function in response to CoPP treatment. However, serum ALT activities remained unchanged at most of the time points investigated. This might have been due to the fact that HO-1 function does not interfere with the underlying liver pathology of *Mdr2*^−/−^ mice based on accumulation of toxic bile salts. Rather, HO-1 induction interferes with subsequent events, such as inflammation and fibrosis^16^ which are known to drive tumour progression^5,27^. An immune inhibitory effect was demonstrated by significantly reduced numbers of CD4^+^ and CD8^+^ T cells in liver tissues of young *Mdr2*^−/−^ mice immediately after HO-1 induction. In contrast, 65-weeks-old tumour bearing *Mdr2*^−/−^ mice showed significantly increased numbers of CD8^+^ T cells in response to early HO-1 induction compared to PBS treated animals, which likely contributes to a more efficient anti-tumour immune response.

In line with that and our previous data^16^, we demonstrated beneficial long-term effects of early HO-1 induction on proliferation and tumour growth. Although CoPP-treated *Mdr2*^−/−^ mice were not completely protected from tumour development, the tumour volume was significantly reduced and delayed due to early HO-1 induction. We observed no effect of early HO-1 induction on formation of dysplastic nodules at the pre-cancerous stage at 48 weeks of age, although CoPP treatment successfully delayed the progression to the malignant state and significantly reduced tumour growth for over two months. We speculate that the equal amount of dyplastic tissue at week 48 is associated with specific features of disease progression in the Mdr2 mouse model. Katzenellenbogen *et al*. demonstrated, that the *Mdr2*^−/−^ phenotype is dominated by a strong inflammatory response in young animals (3 months), which is less pronounced at 12 months of age. Therefore, they concluded that dysplastic nodule formation at 12 months of age is most likely not a direct result of overwhelming inflammatory activity, but of aberrant hepatocyte cell death resulting from either leakage of toxic bile acids or non-direct immune mediated mechanism^5^. Therefore one could speculate that at 48 weeks of age, the anti-inflammatory effect of early HO-1 induction has little effect on the underlying cause of dysplasia. Another possibility is that small differences in dysplastic nodule formation cannot be quantified by histological analysis. Since early CoPP treatment clearly delayed tumour growth in mice older than 48-weeks, we therefore hypothesize that the immune modulatory effect of CoPP is more prominent and allows a more efficient anti-tumour immune surveillance. This is in line with the fact that while 65-week-old PBS treated animals show significantly decreased populations of hepatic CD4^+^ and CD8^+^ T cells, whereas CoPP treated *Mdr2*^−/−^ mice were able to retain almost equal numbers of hepatic T cells over time.

Furthermore, during the early phase of liver disease HO-1 function may alternatively affect regenerative proliferation in *Mdr2*^−/−^ mice through interfering with *Cyclin D1* expression up to 5 months after termination of treatment. This is in line with previous reports of anti-proliferative functions of HO-1 in different mouse models^28^. In addition, we observed HO-1 mediated induction of p38 protein expression and phosphorylation, which may negatively regulate *Cyclin D1* expression up to eight months after CoPP treatment. Chronic regenerative proliferation and exposure to oxidative stress^29^ increases the risk of accumulating mutations and chromosomal aberrations^3^. Once the regenerative capacity is exhausted, chromosome uncapping induces DNA damage signals, senescence, or apoptosis^30^. To our surprise, DNA damage increased over time in F4/80^+^ hepatic macrophages, and much less in hepatocytes. HO-1, as well as its product CO, are known to be essential for DDR after treatment with cytostatic agents or irradiation^31^. Either overexpression of HO-1 or application of CO has been shown to accelerate DDR through phosphorylation of ataxia-telangiectasia mutated protein and downstream repair processes, while the absence of HO-1 results in high levels of DNA damage in various tissues and decreased DDR^31^. We observed a reduced basal *Ho-1* mRNA expression in *Mdr2*^−/−^ mice derived BMDMs and an inability to upregulate *Ho-1* expression as a stress response. This shows that once DNA damage occurs these macrophages cannot initiate DDR via HO-1. DNA damage in hepatic macrophages has been associated with a more pro-inflammatory phenotype^32^. Under physiological and pathophysiological conditions, macrophages are essential for the systemic response to DNA damage. Macrophages have been shown to enhance existing DDR networks and accelerate double strand break repair in neighbouring cells during tissue injury^33^. This is an essential tumour preventing function that might be altered in macrophages with DNA damage.

The involvement of macrophages in all stages of carcinogenesis has been analysed extensively. It has been shown that depending on the microenvironment macrophages can promote malignant transformation in pre-neoplastic lesions, tumour associated neo-angiogenesis, invasion, and metastasis formation^34,35^. Therefore, maintenance of accurate macrophage function is of great importance in regard of tumour development. DNA damage in macrophages is often the result aberrant ROS production^36^. Increased ROS production in macrophages is associated with phagocytic activity, since they are essential for the efficient break down of internalized materials^37^. Another link between DNA damage and phagocytic activity of macrophages has been demonstrated by Pinto *et al*., who showed that irradiated macrophages show increased DNA damage, which led to an increased phagocytic activity and modulated the cytokine production profile of these cells^32^. Furthermore, they could show that while accumulation of DNA damage was associated with a more inflammatory phenotype, these cells retained the ability to promote cancer cell invasion and tumour associated angiogenesis^32^. It can therefore be assumed, that the accumulation of DNA damage in hepatic macrophages contributes to the exacerbation of inflammation in PBS treated *Mdr2*^−/−^ mice, whereas early HO-1 induction reduces inflammation, counteracts ROS production and overall protects genomic stability and functionality of liver macrophages.

Taken together we conclude that early HO-1 induction not only improved chronic inflammation throughout treatment, it mediated anti-inflammatory, anti-tumour effects and DDR in *Mdr2*^−/−^ mice throughout life. We showed that early induction of HO-1 during chronic inflammation lastingly altered the cellular composition of the adaptive immune response. MR imaging confirmed that HO-1 induction delayed tumour growth. Moreover, early induction of HO-1 reduced the expression of *Cyclin D1,* possibly by inducing p38 expression and phosphorylation. On the other hand, HO-1 induction reduced DNA damage in macrophages, which may inhibit their pro-inflammatory phenotype and thereby reduce their tumour promoting function, which overall led to the delayed tumour growth. Collectively these HO-1 mediated effects ameliorated chronic inflammation and delayed tumour development in *Mdr2*^−/−^ mice.

One would not prescribe CoPP as a drug for patients as it contains cobalt, but a variety of approved drugs and compounds induce HO-1 in addition to their pharmacological function^38^. The widely prescribed group of statins as well as curcumin have been shown effective in treating various diseases as well as being effective inducers of HO-1^38^. We therefore conclude it is worth considering HO-1 inducing compounds as an additional therapeutic option in early chronic liver diseases.

## Material and Methods

### Animals

For all experiments female *Mdr2* knockout (*Mdr2*^−/−^; FVB.129P2-Abcb4^tm1Bor^) mice were used, which display a more severe pathology than male mice^39^. FVB/N (WT) mice served as controls. All mice received care according to the FELASA guidelines; the animal protocols were approved by the Hamburg Federal Authority for Health and Environment. Mice were housed in IVC cages under controlled conditions (22 °C, 55% humidity, and 12-hour day-night rhythm) and fed a standard laboratory chow (LASvendi, Soest; Altromin, Lage, Germany).

### HO-1 induction *in vivo*

For the induction of HO-1, *Mdr2*^−/−^ mice were injected intraperitoneally with 5 mg/kg Cobalt protoporphyrin IX (CoPP; Frontier Scientific Europe, Ltd., UK) or PBS twice weekly, for nine weeks (5 to 14 weeks of age). CoPP is a well-established non-substrate inducer of HO-1, proven to be effective *in vitro* and *in vivo*^11^. At the age of either 14, 24, 36, 48 or 65+ weeks mice were sacrificed for analysis. Non-invasive analyses were performed throughout life.

### Determination of plasma transaminase activity and albumin levels

Plasma enzyme activity of alanine aminotransferase (ALT) and plasma levels of albumin were determined as described previously^16^.

### Isolation of Hepatocytes

Hepatocytes were isolated as described previously^40^.

### Isolation of hepatic macrophages

Hepatic non-parenchymal cells were isolated as described previously^41^. F4/80^+^ were enriched by magnetic activated cell sorting using a biotinylated anti-F4/80 antibody (Biolegend; San Diego, CA) and Streptavidin coated magnetic beads (Milteny Biotec, Bergisch Gladbach, Germany).

### Hepatic protein isolation and western blot analysis of p38

Protein lysates were prepared and analysed via western blotting as described previously^16^. Antibodies are summarized in Table 1.

**Table 1:** Antibodies for western blot.

Target	Host	Dilution	Distributed by
p38 MAPK Antibody	rabbit	1:1000	#9212, Cell Signaling, Danvers, MA
Phospho-p38 MAPK Mouse mAb	mouse	1:500	#9216, Cell Signaling, Danvers, MA
Actin (C-11): sc-1615	goat	1:500	#sc-1615, Santa Cruz, Dallas, TX
anti-Rabbit IgG (H + L) HRP	goat	1:5000	#111-035-003, JacksonImmunoResearch, Ely, UK
anti-Mouse IgG2a HRP	horse	1:3000	#7076, Cell Signaling, Danvers, MA
γH2AX	mouse	1:2000	#05-636, Millipore, Burlington, MA
Actin	mouse	1:20000	#A5441, Sigma, St. Louis, MO
IRDye 680RD conj. anti-mouse IgG	goat	1:7500	#925-68070, Licor, Lincoln, NE

MAPK: mitogen-activated protein kinase; IgG: immunoglobulin G, HRP: horseradish peroxidase, γH2AX: phosphorylated histone H2AX.

### Western blot analysis of γH2AX in hepatocytes and hepatic macrophages

Total protein was extracted from isolated hepatocytes and hepatic macrophages. 40 µg of total protein were resolved by SDS-PAGE using a 4–15% gradient gel (Bio-Rad, Hercules, CA). After transfer to a Nitrocellulose membrane (Licor, Lincol, NE), proteins were detected by Anti-phospho-Histone H2A.X (Ser139) Antibody, (1:2000; Millipore Burligton, MA), and anti-β-actin (Sigma, 1:20.000) and IRDYE 680 conjugated anti-mouse IgG (Licor, Lincol, NE; 1:7.500) was used. Antibodies are summarized in Table 1.

### Analysis of mRNA expression

Isolation of total RNA, cDNA synthesis and real time RT-PCR were carried out as described previously^42^. Oligonucleotides are summarized in Table 2.

**Table 2:** Oligonucleotides for real time RT-PCR.

Target	Forward primer	Reverse primer	Reference
*Cd36*	TCGGATCTGAAATCGACCTT	CACAGGCTTTCCTTCTTTGC	NM_001159557.1
*CyclinD1*	AGTGCGTGCAGAAGGAGATT	CACAACTTCTCGGCAGTCAA	NM007631
*Ho-1*	GAGATAGAGCGCAACAAGCA	CTTGACCTCAGGTGTCATCTC	NM010442

*Cd*: cluster of differentiation; *Ho-1*: heme oxygenase 1.

### Immunohistochemistry and H&E staining

Liver and tumour tissue was processed as described previously^16^. Cryo sections were processed as described previously^43^. Antibodies are summarized in Table 3. Routine hematoxylin & eosin staining of tumour tissue was performed for histological tumour typing and grading by a pathologist.

**Table 3:** Antibodies for immunohistochemistry.

Target	Host	Dilution	Distributed by
F4/80	rat	1:200	#MCA497R, Bio Rad, Hercules, CA
HNF4-alpha	goat	1:100	#sc-6556, Santa Cruz, Dallas, TX
γH2A.X	rabbit	1:200	#2577, Cell Signaling, Danvers, MA
anti-Rabbit IgG (H + L) Alexa Fluor 488	goat	1:200	#A-11008, Life Technologies Carlsbad, CA
anti-Rat IgG (H + L) Alexa Fluor 594	donkey	1:200	#A-21209, Life Technologies, Carlsbad, CA
anti-Phospho-Histone H2A.x	mouse	1:200	#05-636, Millipore, Burlington, MA
anti-mouse ALEXA fluor 594	goat	1:600	#A-11005 Thermo Fisher, Waltham, MA

HNF4: hepatocyte nuclear factor 4; γH2AX: phosphorylated histone H2AX; IgG: immunoglobulin.

### Flow Cytometry

Flow cytometric analysis was performed as described previously^41^. Antibodies are summarized in Table 4.

**Table 4:** Antibodies for flow cytometry.

Target	Conjugate	Dilution	Clone	Distributed by
CD19	Brilliant Violet 785	1:200	6D5	BioLegend, San Diego, CA
CD3	Brilliant Violet 650	1:200	17A2	BioLegend, San Diego, CA
CD8a	FITC	1:200	53–67	BD Pharmingen, San Jose, CA
CD4	Brilliant Violet 711	1:500	RM4–5	BioLegend, San Diego, CA
NK1.1	PE	1:200	PK 136	BioLegend, San Diego, CA
Foxp3	PE-Cy5	1:100	FJK165	eBioscience, San Diego, CA

CD: cluster of differentiation; FITC: fluorescein isothiocyanate; Foxp3: forkhead-box P3.

### MR Imaging

The MRI dataset consists of 1305 measurements for 87 subjects over 30 weeks (15 measuring points). Measurements were taken every other week, from approximately 38 weeks of age throughout life, using a T2-weighted turbo spin echo sequence, as described previously^44^.

### Image Analysis

Quantitative image analysis was performed using the open source software ImageJ (National Institutes of Health, Bethesda, MD) and qMapIt plugin^45^. Regions of interest were manually placed and tumour volume of each individual tumour was measured.

### Irradiation

Cells were irradiated at room temperature with 200 kV X-rays (Gulmay RS225, Gulmay Medical Ltd., Byfleet, UK; 15 mA, 0.8 mm Be + 0.5 mm Cu filtering; dose rate of 1.2 Gy/min).

### Bone marrow derived macrophages

Bone marrow derived macrophages were differentiated according to a protocol adapted from Weischenfeldt *et al*.^46^. In brief, bone marrow cells were isolated from the tibia and femur. Cells were washed with PBS and cultured on tissue culture plates in RPMI medium containing 1% penicillin/streptomycin, 20% FCS, and 30% supernatant of L929 cells. BMDMs were incubated for 4 days; medium was changed after 46 h. Cells were treated with or without CoPP [10 µg/ml] for 24 h prior to further procedures.

### Phagocytosis Assay

BMDMs (2 × 10^5^) were cultured with 5 × 10^5^ APC^+^ BD Calibrite beads™ (BD bioscience, Franklin Lake, NJ) for 4 h at 37 °C. Cells were subsequently fixated in 2% PFA solution and analysed with a BD FACSCanto™ II system (BD bioscience, Franklin Lake, NJ).

### Immunofluorescence

Immunofluorescence staining of γH2AX was performed as described previously^47^. Antibodies are summarized in Table 3.

### Statistical analysis

Data sets were analysed using either a student’s t test with post-hoc Welch’s correction or a Mann-Whitney test if two groups were compared. If three or more groups were compared a one-way ANOVA with Bonferroni post-test was used. For frequency distribution analysis, a paired t test with a Wilcoxon’s post-test were used. All data in this study are expressed as mean +/− SEM. *P ≤ 0.05, **P ≤ 0.01, ***P ≤ 0.001, ****P ≤ 0.0001.

The datasets generated during and/or analysed during the current study are available from the corresponding author on reasonable request.

## Electronic supplementary material

Supplementary Figures
