# Supplementary material for: Early heme oxygenase 1 induction delays tumour initiation and enhances DNA damage repair in liver macrophages of Mdr2−/− mice
Source: Sci Rep. 2018 Nov 2;8:16238. doi: 10.1038/s41598-018-33233-0 (PMC6214975; doi:10.1038/s41598-018-33233-0)
Supplement: Supplementary file 1 — Supplementary Figures [file 41598_2018_33233_MOESM1_ESM.pdf]

Early heme oxygenase 1 induction delays tumor initiation and enhances DNA damage repair in liver macrophages of *Mdr2*$^{-/-}$ mice

Roja Barikbin, Laura Berkhout, Julia Bolik, Dirk Schmidt-Arras, Thomas Ernst, Harald Ittrich, Gerhard Adam, Ann Parplys, Christian Casar, Till Krech, Khalil Karimi, Gabriele Sass, Gisa Tiegs

Supplementary Images

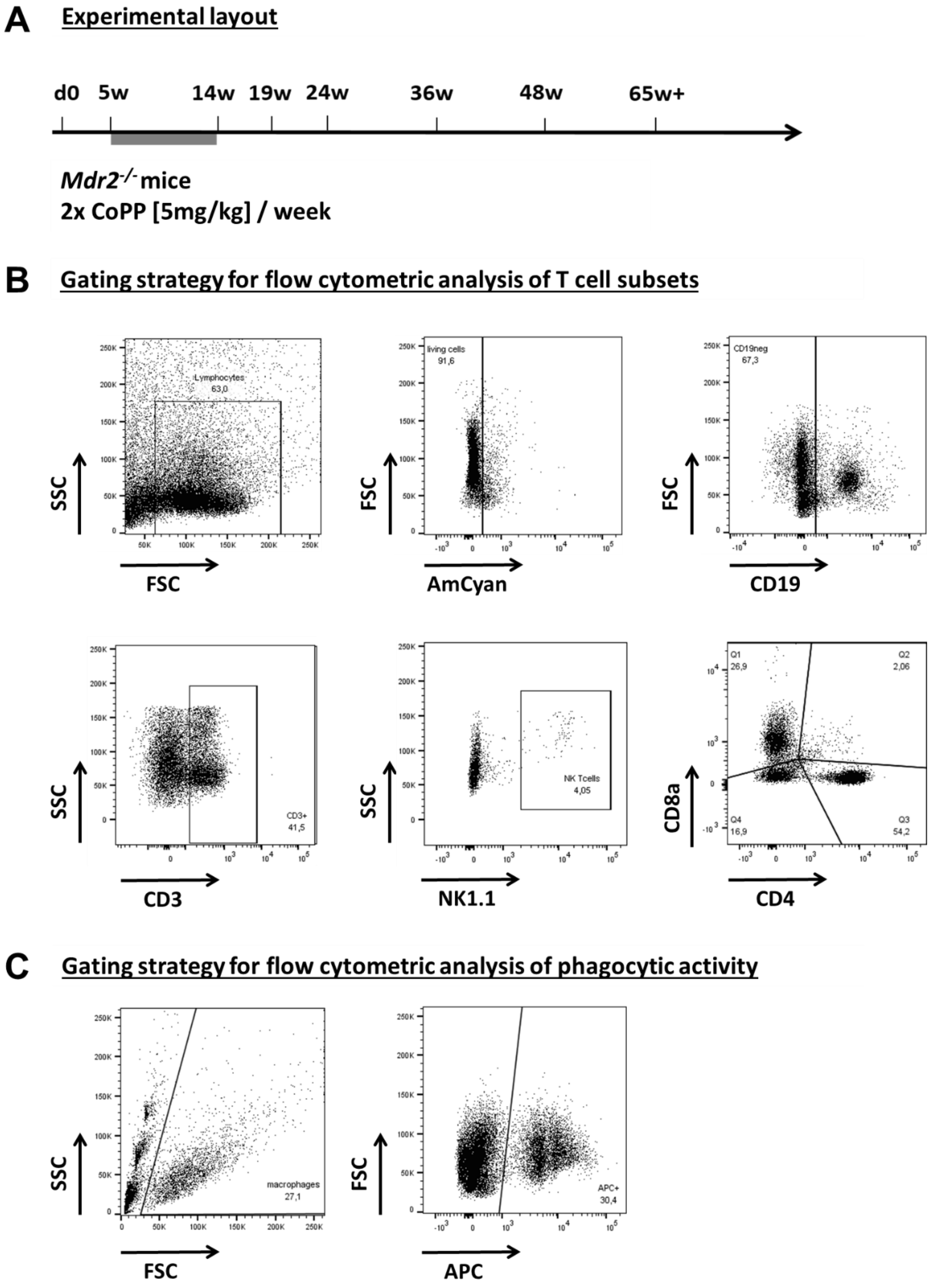

Supplementary Figure 1: (A) Experimental layout: *Mdr2*$^{-/-}$ mice were treated with HO-1 inducer CoPP [5 mg/kg] twice a week for nine consecutive weeks (week 5-14). At week 14, 24, 36, 48 or 65+

mice were sacrificed for analysis. (B) Gating strategy for T cell subsets**:** Forward / sideward scatter and AmCyan staining determined vital total leucocytes. $CD19^-$ and $CD3^+$ cells were further analysed for $NK1.1^+$ NKT cells, $CD4^+$ T cells, and $CD8^+$ T cells. (C) Gating strategy for the phagocytosis assay of bone marrow derived macrophages with APC^+ BD Calibrite bead (BD Pharmingen, San Jose, CA): Macrophages were identified via forward / sideward scatter. Phagocytic macrophages were identified via APC fluorescence of engulfed beads.

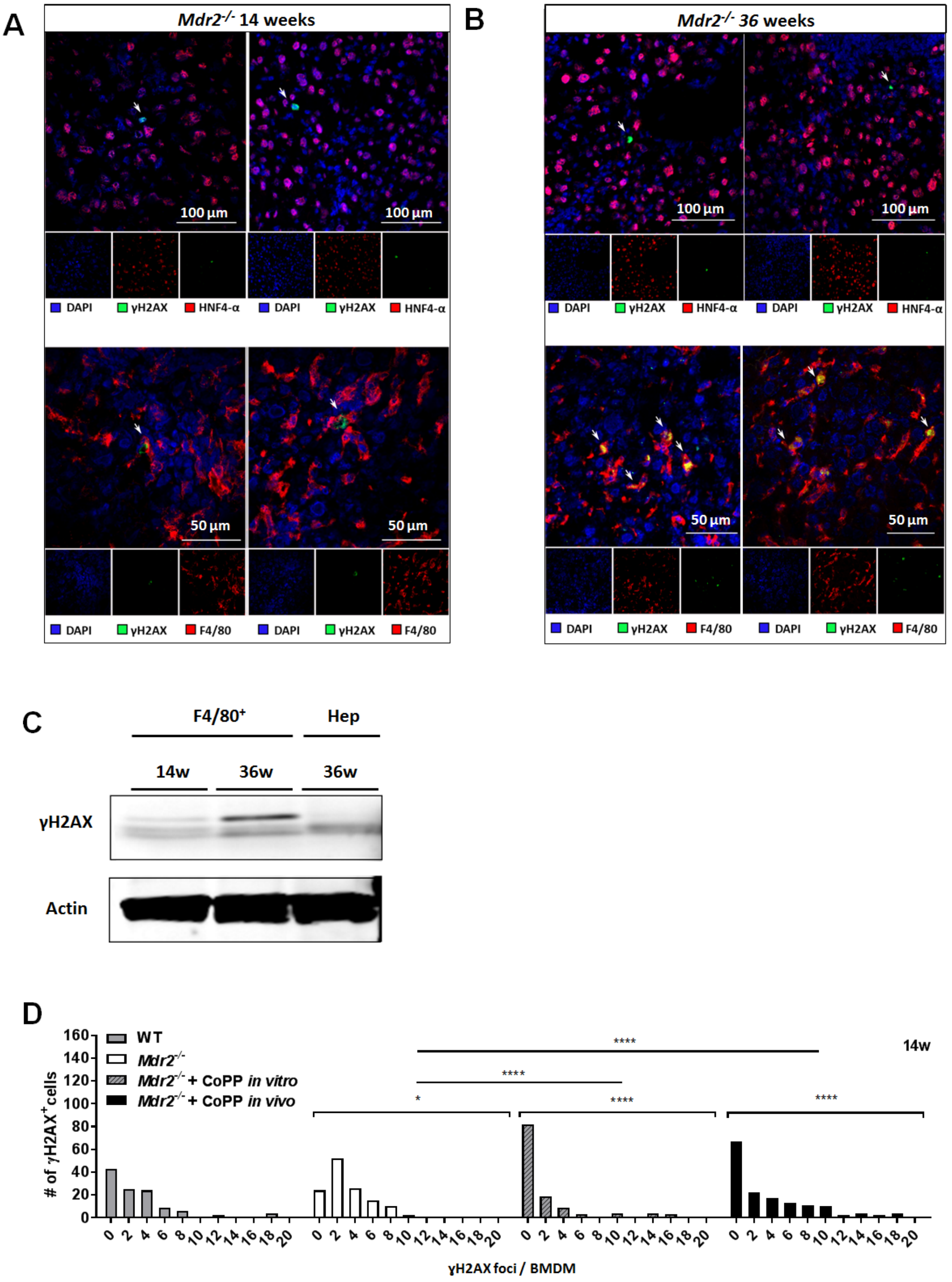

Supplementary Figure 2: (A) Representative images (20x) of tissue sections of 14- and (B) 36-week-old *Mdr2*$^{-/-}$ mice stained for DAPI, γH2AX, and the hepatocyte marker HNF4-α (upper panel) as well as DAPI, γH2AX, and the macrophage marker F4/80 (lower panel). (C) Protein levels of γH2AX in F4/80^{+} macrophages and hepatocytes isolated from in 14- and 36-week old *Mdr2*$^{-/-}$ mice. The full length western blot is presented in Suppl. Figure 4B. (D) Frequency distribution of γH2AX^{+} foci in BMDMs (14-weeks-old; WT, *Mdr2*$^{-/-}$) with or without CoPP treatment *in vitro* [10µg/ml; 24h] or *in vivo* as described in Suppl. Figure 1A.

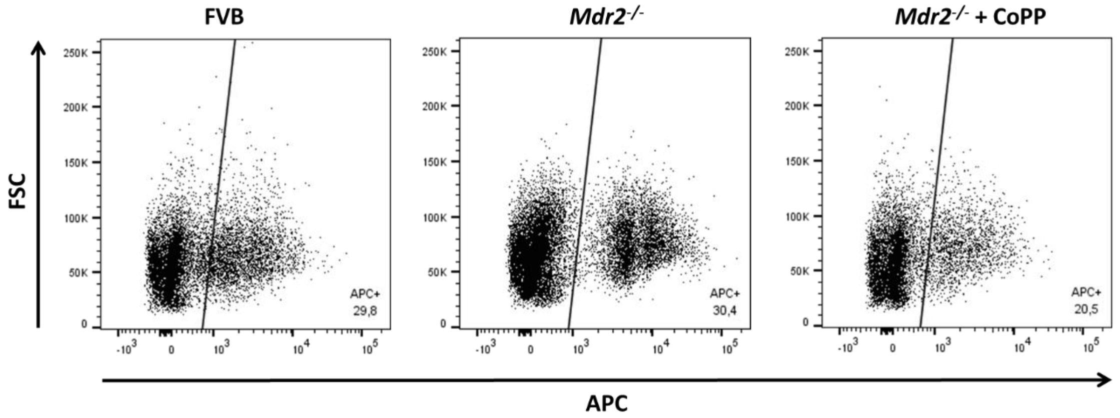

Supplementary Figure 3: Representative dot plots of the phagocytosis assay presented in Figure 4G. Bone marrow derived macrophages of FVB (n = 3) and *Mdr2*$^{-/-}$ mice treated with PBS (n = 3) or 10 µg/ml CoPP (n = 3) for 24 h prior to incubation with APC^{+} BD Calibrite bead (BD Pharmingen, San Jose, CA) for 4 h at 37 °C.

A **Full length western blots of p38, p-p38 and actin as loading control**

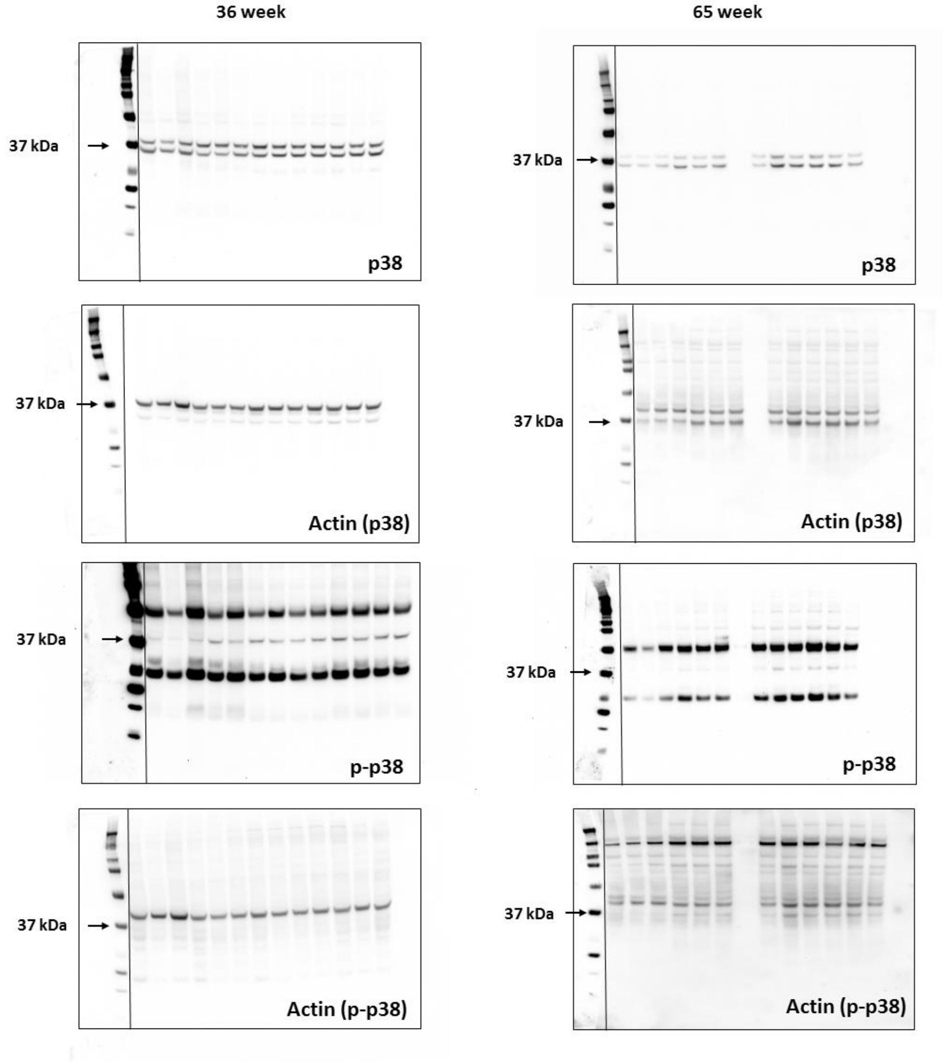

B **Full length western blot of γH2AX and actin as loading control**

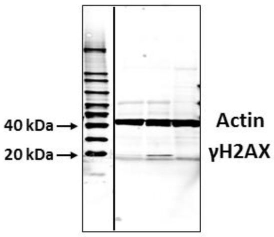

Supplementary Figure 4: (A) Full length western blot of Figure 3B (left) and 3D (right). The liver tissue lysates for p38 and p-p38 WBs were derived from the same experiment and gels/blots were processed in parallel. The molecular marker (Precision Plus Protein WesternC Standards) was loaded onto the same gel and processed with the samples. However, the standard was cut from the blot prior to development with Strep-Tactin HRP (iba) in order to prevent unspecific biding of Strep-Tactin to the remaining blot. Gels were loaded as follows, (left) Lane 1: marker, Lane 2-4: WT, Lane 5-9: *Mdr2*$^{-/-}$, Lane 10-14: Mdr2$^{-/-}$ + CoPP. (Right) Lane 1: marker, Lane 2-4: WT, Lane 5-7;9: Mdr2$^{-/-}$, Lane 8: empty, Lane 10-14: Mdr2$^{-/-}$ + CoPP. Each lane represents one animal. (B) Full length blot of Suppl. Figure 2C. Protein lysates were prepared from isolated F4/80^{+} macrophages and hepatocytes and run at the same time. The molecular marker (MagicMark XP Western Protein Standard) was moved from the right to the left side. The gel was loaded as follows, Lane 1: F4/80^{+} cells isolated and pooled from 14-week-old Mdr2$^{-/-}$ mice (n=3), Lane 2: F4/80^{+} cells isolated and pooled from 36-week-old Mdr2$^{-/-}$ mice (n=4), Lane 3: Hepatocytes isolated from a Mdr2$^{-/-}$ mouse, Lane 4: Molecular marker

(Seehorse, not shown). (Lane 5-17: not shown), Lane 18: Molecular marker (MagicMark XP Western Protein Standard; moved to the left).
